# Supplementary material for: Exogenous GA3 Enhances Nitrogen Uptake and Metabolism under Low Nitrate Conditions in ‘Duli’ (Pyrus betulifolia Bunge) Seedlings
Source: Int J Mol Sci. 2024 Jul 21;25(14):7967. doi: 10.3390/ijms25147967 (PMC11277063; doi:10.3390/ijms25147967)
Supplement: Supplementary file 1 [file ijms-25-07967-s001.zip › ijms-3083001-supplementary.pdf]

Exogenous GA_3 Enhances Nitrogen Uptake and Metabolism under Low Nitrate Conditions in 'Duli' (*Pyrus betulifolia* Bunge) Seedlings

Weilong Zhang, Xiaohua Cheng, Zhaotian Jing, Ying Cao, Shuai Yuan, Haixia Zhang and Yuxing Zhang *

College of Horticulture, Hebei Agricultural University, Baoding 071001, China; 18322711826@163.com (W.Z.); chengxiaohua2016@126.com (X.C.); 13409298238@163.com (S.Y.); zhx2323a@163.com (H.Z.)

* Correspondence: zhyx@hebau.edu.cn

Supplementary Data

Table S1 Effects of GA_3 and PAC on the growth, chlorophyll content and root length of 'Duli'. Data are presented as means ± SD (n = 3). Values not followed by the same letter denote significant differences based on Tukey's multiple-range tests ($P < 0.05$).

Concentration (mM)	Plant length	Leaf number	Leaf area per plant	Chlorophyll content	Main root length
CK	8.19±0.52c	7.40±0.52a	2.38±0.08b	2.21±0.04e	17.85±0.38cd
0	7.68±0.33d	6.60±0.52bc	1.910±0.07e	1.97±0.07g	18.21±0.45cd
GA_3 (0.01)	7.82±0.34d	6.40±0.52bc	1.96±0.05e	1.94±0.02g	18.38±0.34bc
GA_3 (0.05)	8.28±0.25c	6.80±0.42b	2.16±0.09d	1.98±0.05fg	18.91±0.49b
GA_3 (0.1)	9.44±0.34a	7.40±0.84a	2.61±0.13a	2.04±0.05fg	19.82±0.70a
GA_3 (0.15)	8.54±0.30b	7.60±0.52a	2.27±0.13c	2.10±0.05ef	18.92±0.53b
PAC (0.005)	7.70±0.09d	6.20±0.42cd	1.90±0.06e	2.73±0.09c	18.11±1.08cd
PAC (0.01)	7.31±0.10e	5.80±0.42de	1.55±0.04f	3.17±0.05a	17.66±0.85de
PAC (0.02)	7.06±0.14e	5.60±0.52e	1.30±0.08	3.00±0.09b	17.16±0.40e
PAC (0.04)	6.73±0.26f	5.40±0.52e	0.94±0.07h	2.92±0.06b	14.76±0.41f
PAC (0.1)	6.49±0.22f	5.30±0.48e	0.59±0.05i	2.48±0.10d	14.64±0.27f

Table S2 The membership function values and comprehensive evaluation of different GA_3/PAC concentration.

Concentrati on (mM)	Plant length	Leaf numbe r	Leaf area per plant	Chloroph yll content	Main root length	Comprehen sive assessment	Rankin g
CK	0.58	0.91	0.89	0.22	0.62	0.64	3
0	0.45	0.48	0.74	0.00	0.72	0.48	8
GA_3 (0.01)	0.45	0.48	0.74	0.00	0.72	0.48	8
GA_3 (0.05)	0.61	0.65	0.83	0.03	0.82	0.59	6
GA_3 (0.1)	1.00	0.91	1.00	0.08	1.00	0.80	1
GA_3 (0.15)	0.69	1.00	0.85	0.13	0.83	0.70	2
PAC (0.005)	0.59	0.39	0.71	0.64	0.67	0.60	5
PAC (0.01)	0.72	0.22	0.57	1.00	0.58	0.62	4
PAC (0.02)	0.81	0.13	0.47	0.86	0.49	0.55	7
PAC (0.04)	0.92	0.04	0.35	0.80	0.02	0.43	10

PAC (0.1)	1.00	0.00	0.00	0.44	0.00	0.29	11

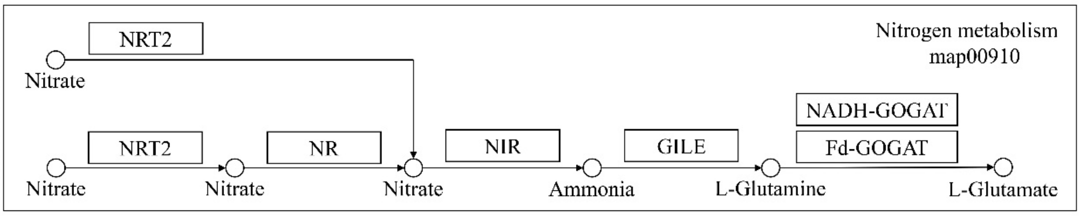

Figure S1. Map of key gene pathways involved in N uptake and metabolism.

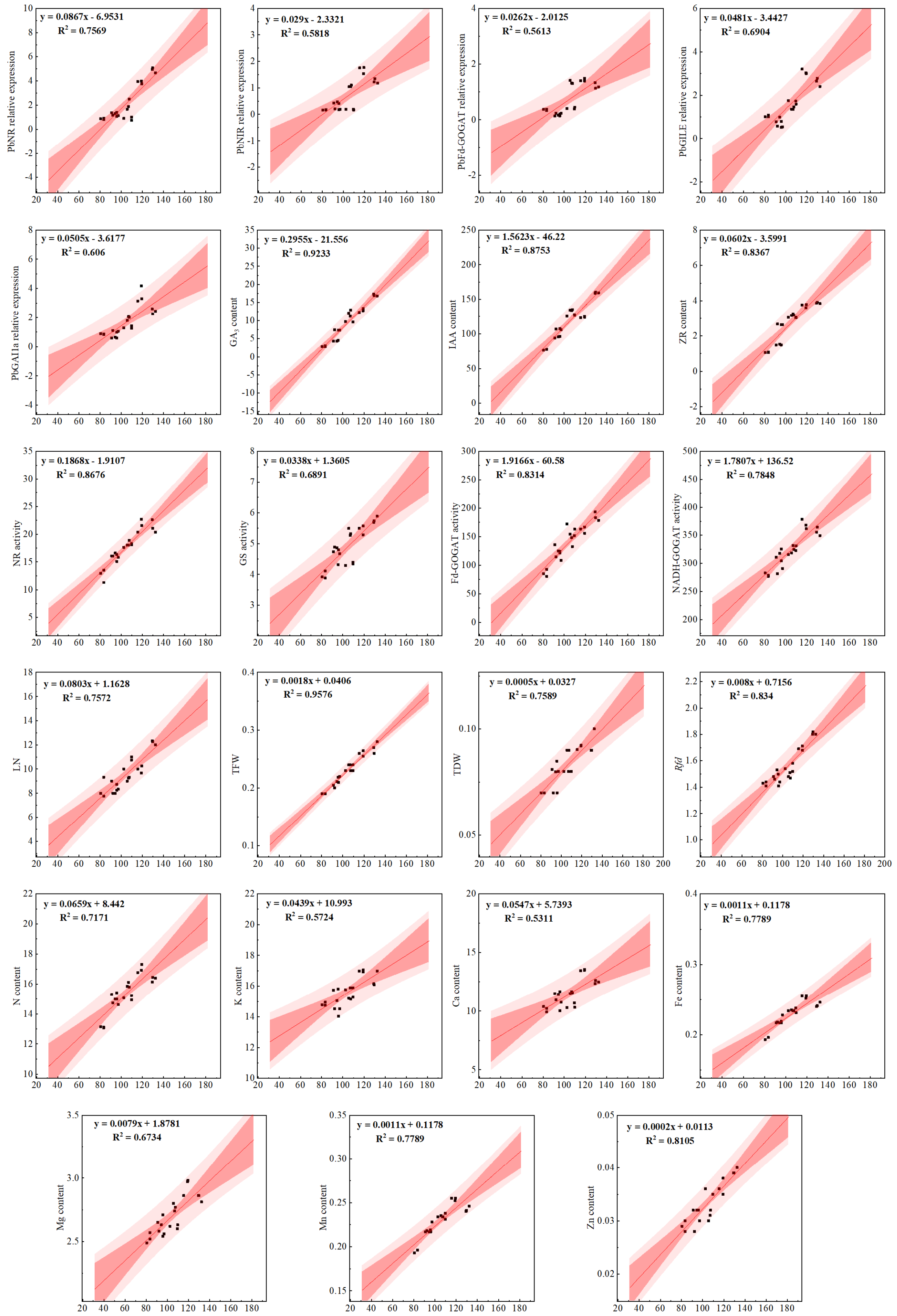

Figure S2. Relationships between the aboveground index and plant length (Pearson's correlation analysis). Only the aboveground indices that are significantly correlated with plant length are shown in the figure.

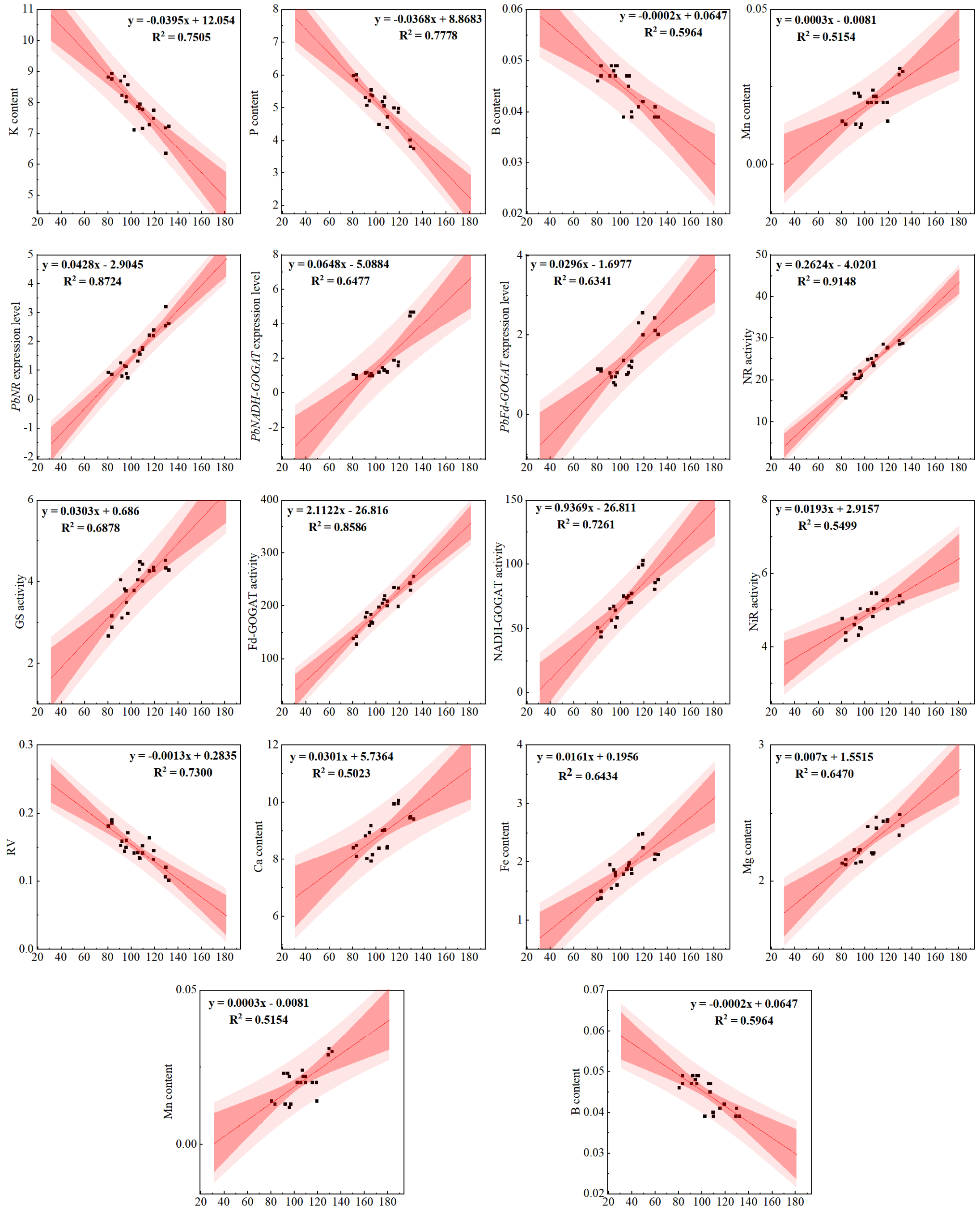

Figure S3. Relationships between the root index and plant length (Pearson's correlation analysis). Only the root indices that are significantly correlated with plant length are shown in the figure.

Table S3 Comprehensive score of exogenous GA_3 in 'Duli' under NO_3^- deficiency. SCK, 0.5 mM NO_3^- solution; SGT, 0.5 mM NO_3^- solution with 0.1 mM GA_3; SPT, 0.5 mM NO_3^- solution with 0.01 mM PAC; DCK, 8 mM NO_3^- solution; DGT, 8 mM NO_3^- solution with 0.1 mM GA_3; DPT, 8 mM NO_3^- solution with 0.01 mM PAC; CK, 16mM NO_3^- solution.

**Treatment**	**PC1**	**PC2**	**PC3**	**PC4**	**PC5**	**Comprehensive score**	**Ranking**
SCK	-0.19	-0.34	-0.07	0.02	0.02	-0.55	6
SGT	0.10	-0.35	0.04	-0.02	-0.04	-0.27	5
SPT	-0.98	0.04	0.01	-0.06	0.02	-0.96	7
DCK	0.31	0.05	-0.03	0.08	0.01	0.43	3
DGT	0.50	0.09	0.17	-0.01	0.01	0.77	2
DPT	-0.57	0.34	-0.02	0.04	-0.03	-0.25	4
CK	0.81	0.17	-0.11	-0.06	0.00	0.82	1

Table S4 The concentrations of nutrition solution and allogenic material of each treatment. SCK, 0.5 mM NO_3^- solution; SGT, 0.5 mM NO_3^- solution with 0.1 mM GA_3; SPT, 0.5 mM NO_3^- solution with 0.01 mM PAC; DCK, 8 mM NO_3^- solution; DGT, 8 mM NO_3^- solution with 0.1 mM GA_3; DPT, 8 mM NO_3^- solution with 0.01 mM PAC; CK, 16 mM NO_3^- solution.

**Composition**		**Purity (≥%)**	**SCK**	**SGT**	**SPT**	**DCK**	**DGT**	**DPT**	**CK**
Macroelement (mM)	KH_2PO_4	99.50	1.00	1.00	1.00	1.00	1.00	1.00	1.00
	$MgSO_4 \cdot 7H_2O$	99.00	2.00	2.00	2.00	2.00	2.00	2.00	2.00
	KCl	99.50	5.00	5.00	5.00	5.00	5.00	5.00	5.00
	$Ca(NO_3)_2$	98.50	0.50	0.50	0.50	8.00	8.00	8.00	16.00
	$CaCl_2$	96.00	7.50	7.50	7.50	8.00	8.00	8.00	0.00
Microelement (mg/L)	KI	99.00	0.83	0.83	0.83	0.83	0.83	0.83	0.83
	H_3BO_3	99.50	6.20	6.20	6.20	6.20	6.20	6.20	6.20
	$ZnSO_4$	99.50	8.60	8.60	8.60	8.60	8.60	8.60	8.60
	Na_2MoO_4	99.00	0.25	0.25	0.25	0.25	0.25	0.25	0.25
	$CuSO_4$	99.00	0.03	0.03	0.03	0.03	0.03	0.03	0.03
	$CoCl_2$	99.00	0.03	0.03	0.03	0.03	0.03	0.03	0.03
	$MnSO_4$	99.00	22.30	22.30	22.30	22.30	22.30	22.30	22.30
Iron salt (mM)	EDTA-Fe	99.50	0.05	0.05	0.05	0.05	0.05	0.05	0.05
Allogenic material(mM)	PAC	95.00	0	0	0.01	0	0	0.01	0
	GA_3	99.00	0	0.10	0	0	0.10	0	0

Table S5 Sequences of primers used in qRT-PCR.

**Gene**	**Primer sequence (5'-3')**
PbACTIN F	TTGGTATGGGTCAGAGG
PbACTIN R	CTGTGAGCAGAACTGGGTG
PbNRT2 F	AAGATCATCGTGGTATGTTTGC
PbNRT2 R	GAAGGTGGAGAAGAAGTTGGAG

PbNR F	CCCACTCTGCGACTTGCTTA
PbNR R	AACCACTTCACCATCCGACC
PbNiR F	GCATTGACCCCCTCGAGATT
PbNiR R	TGAATCCGAACCTGCCATCC
PbNADH-GOGAT F	TAGAGGTTGGCGCTGCATAG
PbNADH-GOGAT R	CCACTTGTGTGCGTTCAAGG
PbFd-GOGAT F	CGGACTCTTCGTCGTCGATT
PbFd-GOGAT R	AGTGGCAATTCCTGGAGACG
PbGILE F	AGCAAAGCAAGGACTCTGCC
PbGILE R	AGCCCTCTTGTTGGTTGGAA
PbGAI1a F	TCCCTCCGAACTCCTCTT
PbGAI1a R	GTAGCCGATCTGGGTCATC
